# Bioengineering for vascularization: Trends and directions of photocrosslinkable gelatin methacrylate hydrogels

**DOI:** 10.3389/fbioe.2022.1053491

**Published:** 2022-11-17

**Authors:** Gwang-Bum Im, Ruei-Zeng Lin

**Affiliations:** ^1^ Department of Cardiac Surgery, Boston Children’s Hospital, Boston, MA, United States; ^2^ Department of Surgery, Harvard Medical School, Boston, MA, United States

**Keywords:** vascularization, endothelial cells, regenerative medicine, tissue engineering, gelatin methacrylate (GelMA)

## Abstract

Gelatin methacrylate (GelMA) hydrogels have been widely used in various biomedical applications, especially in tissue engineering and regenerative medicine, for their excellent biocompatibility and biodegradability. GelMA crosslinks to form a hydrogel when exposed to light irradiation in the presence of photoinitiators. The mechanical characteristics of GelMA hydrogels are highly tunable by changing the crosslinking conditions, including the GelMA polymer concentration, degree of methacrylation, light wavelength and intensity, and light exposure time et al. In this regard, GelMA hydrogels can be adjusted to closely resemble the native extracellular matrix (ECM) properties for the specific functions of target tissues. Therefore, this review focuses on the applications of GelMA hydrogels for bioengineering human vascular networks *in vitro* and *in vivo*. Since most tissues require vasculature to provide nutrients and oxygen to individual cells, timely vascularization is critical to the success of tissue- and cell-based therapies. Recent research has demonstrated the robust formation of human vascular networks by embedding human vascular endothelial cells and perivascular mesenchymal cells in GelMA hydrogels. Vascular cell-laden GelMA hydrogels can be microfabricated using different methodologies and integrated with microfluidic devices to generate a vasculature-on-a-chip system for disease modeling or drug screening. Bioengineered vascular networks can also serve as build-in vasculature to ensure the adequate oxygenation of thick tissue-engineered constructs. Meanwhile, several reports used GelMA hydrogels as implantable materials to deliver therapeutic cells aiming to rebuild the vasculature in ischemic wounds for repairing tissue injuries. Here, we intend to reveal present work trends and provide new insights into the development of clinically relevant applications based on vascularized GelMA hydrogels.

## Introduction

The majority of tissues in the body require blood flow to provide nutrients and oxygen to individual cells (Rouwkema et al., 2008). Because the diffusion limit of oxygen through biological tissues is around 100–200 μm, proximity to the capillary is essential for cell survival (Novosel et al., 2011). The vascular densities are even higher in high-metabolic-rate organs, such as hearts and livers. In these organs, approximately every parenchymal cell is in direct contact with at least one microvessel to meet the metabolic demand (Lorente et al., 2020). Vascular disorders can occur at any level of the hierarchical vascular network (Udan et al., 2013). Genetic defects in critical genes of vascular development generally result in early embryonic lethal (Park et al., 2013). In adults, diminished or absent blood flow causes ischemic injury, resulting in the inability to maintain cell viability, build-up of metabolic waste products, and eventual leakage of proteolytic enzymes into the surrounding tissues (Katarzyna, 2017; Qadura et al., 2018; Hess et al., 2019). Ischemic injury happening in vital organs immediately leads to life-threatening diseases. The causes of ischemic injury vary widely in different conditions. For example, coronary artery disease is caused by narrowed coronary arteries which supply blood to the heart muscle (Nowbar et al., 2019). Ischemic stroke occurs when a blood clot blocks or narrows an artery leading to the brain (Tsao et al., 2022). Chronic metabolic disorders and aging generally increase the risk of vascular disorders, including congestive heart failure, stroke, critical limb ischemia, and diabetic-related retinopathy and nephropathy (Boudina and Abel, 2007; Dokken, 2008; Assar et al., 2016; Tsao et al., 2022). In the United States, vascular disorders lead to severe complications of cardiovascular diseases, which have remained the leading cause of mortality (Tsao et al., 2022).

Ischemic diseases caused by the blockage of major arteries are predominately treated by surgical bypass interventions, mainly using vascular grafts to revascularize downstream tissues (Zeltsman and Acker, 2002). Notwithstanding the benefit produced by these surgical procedures, inadequate revascularization remains a common outcome due to the inability to regenerate microvascular beds in the ischemic areas (Carmeliet and Jain, 2011). For years, considerable effort has been focused on delivering pro-angiogenic growth factors, genes, and vascular progenitor cells to promote local revascularization (Rafii and Lyden, 2003; Carmeliet and Jain, 2011). These strategies recently gained significant progression from the interdisciplinary knowledge of stem cell biology and biomaterial engineering (Rouwkema et al., 2008; Lovett et al., 2009; O’Connor et al., 2022).

Tissue engineering holds great promise in regenerative medicine as a means to generate competent tissues that can be transplanted to replace damaged body parts. However, despite remarkable pre-clinical progress, translation of tissue engineering products into clinical practice still faces a myriad of difficulties. One major challenge is the necessity to integrate complex three-dimensional. (3D) vascular networks into bioengineered constructs to sustain the transplantation of engineered tissues (Rouwkema et al., 2008; Lovett et al., 2009; Wang et al., 2019). Avascular tissue-engineered constructs are very likely to struggle after *in vivo* transplantation due to limited oxygen and nutrient supply. Studies have consistently shown that the ingrowth of pre-existing host microvessels is insufficient to ensure appropriate vascularization of implanted tissues (Rademakers et al., 2019). To achieve rapid and complete vascularization of thick engineered tissues, constructs would need some kind of built-in vasculature (Novosel et al., 2011).

Cell transplantation also requires a strategy to ensure adequate oxygenation, nutrient delivery, and removal of waste products to achieve successful cell engraftment. Injection of several therapeutic cell types, including the induced pluripotent stem cell (iPSC)-derived cardiomyocytes, hepatocytes, and pancreatic beta cells, have been shown to survive and function significantly better with built-in vasculature (Takebe et al., 2013; Chong et al., 2014; Vlahos et al., 2017). Over the last two decades, researchers have resorted to exploiting the inherent blood vessel-forming ability of vascular progenitor cells in an effort to incorporate such built-in vascular networks (Melero-Martin et al., 2007; Wang et al., 2019). The options for clinically available human vascular progenitor cells were covered in our previous review article (Wang et al., 2019). Currently, consensus still holds that bioengineering vascular networks remain a priority in cell- and tissue-based regenerative medicine. The use of proper biomaterials as a vehicle to facilitate vasculogenesis is central to this effort.

This review paper aims to provide a comprehensive overview of the recent progress in utilizing gelatin methacrylate (GelMA) for bioengineering human vascular networks. We cover the advantages of GelMA compared to other natural or synthetic hydrogels regarding the compatibility of vessel formation. GelMA-based *in vitro* and *in vivo* strategies to construct functional vascular networks are also reviewed with a particular interest in their therapeutic applications.

## Synthesis and preparation of gelatin methacrylate

**FIGURE 1:**
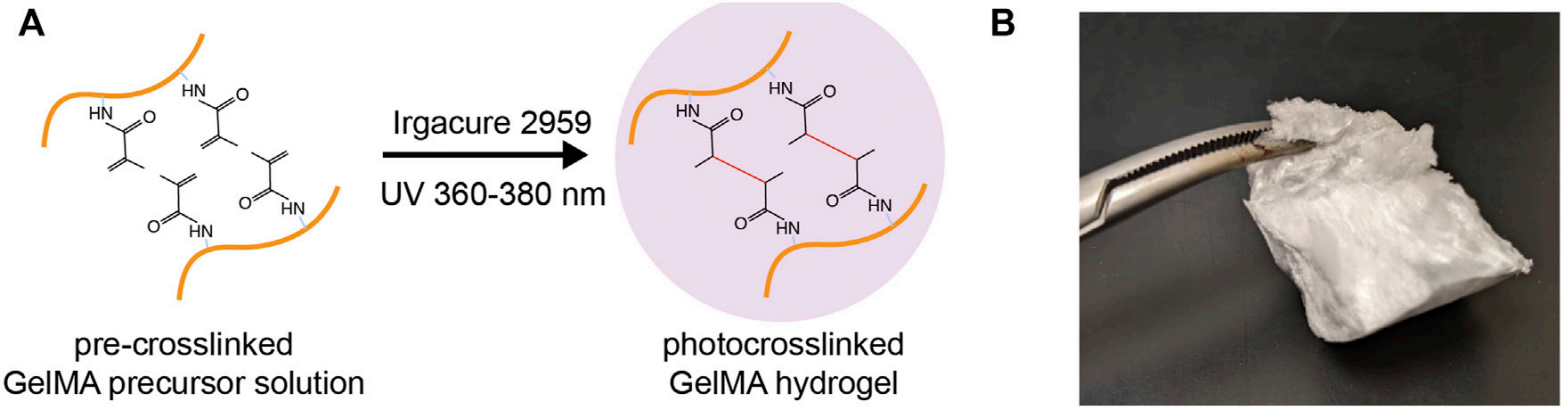
Gelatin methacrylate (GelMA). **(A)** Schematic depicting the photocrosslinking of GelMA from a precursor solution to a hydrogel matrix in the presence of photoinitiators and UV irradiation. **(B)** A piece of freeze-lyophilized GelMA foam.

GelMA is modified from gelatin, the hydrolyzed product of collagen at high temperatures. GelMA is synthesized by adding methacrylate groups to the amine-containing side-groups of gelatin, which becomes a photocrosslinkable biopolymer (Figure 1) (Nichol et al., 2010; Loessner et al., 2016). This reaction is achieved by adding methacrylic anhydride (MA) dropwise into a gelatin solution under vigorous stirring. Final concentrations of MA between 1 and 10% (v/v) are commonly used in reported studies. Higher MA concentration results in a higher degree of methacrylation (defined as the ratio of functionalized to original amino groups and measured by ^1^H-NMR spectroscop) (Chen et al., 2012). Unreacted MA and additional by-products are removed by dialysis against deionized water using 12–14 kDa cut-off dialysis tubes. The dialyzed GelMA solution is freeze-lyophilized to form a foam (Figure 1B).

The usage of GelMA starts with the preparation of GelMA precursor solution. Lyophilized GelMA foam can be dissolved in warm biological buffers (i.e., PBS, normal saline, and cell culture media) to a concentration of up to 20% (w/v). This GelMA precursor solution is stable at room temperature for several weeks. GelMA crosslinking occurs in the presence of photoinitiators and the irritation of visible or ultraviolet (UV) light matching the photoinitiators (Figure 1A). Common photoinitiators compatible with biological applications are Irgacure 2959 (for UV light) and Lithium phenyl-2,4,6-trimethylbenzoylphosphinate (LAP; for visible light; absorbance at 400 nm) (Sharifi et al., 2021).

The mechanical property, water retention, and degradability of GelMA hydrogel are highly tunable (Loessner et al., 2016). In general, the usage of GelMA with a higher methacrylate degree and the higher GelMA polymer concentration in the precursor solution produce a stiff hydrogel with a slow degradation rate. A higher light illumination intensity also achieves a stronger crosslinked GelMA hydrogel. Other crucial parameters for controlling photocrosslinking include the choice of photoinitiators, the light wavelength and intensity, the distance and the presence of biological cells/tissues between the light source and GelMA solution, and the overall exposure time. In practice, we suggest titrating the light exposure time to optimize the GelMA hydrogel properties while keeping the other parameters constant.

For cell-laden GelMA hydrogels, the cell viability is significantly influenced by the choice of photocrosslinking conditions and is highly dependent on the cell types. Overcrosslinked GelMA hydrogels significantly impair vascular morphogenesis (Chen et al., 2012; Lin et al., 2013). We observed several critical steps of vascularization are compromised if the matrix is too stiff, including the loss of vascular cell viability, the failure of cell spreading and migration, and the lack of host-graft cellular interactions in general (Chen et al., 2012; Lin et al., 2013; Chuang et al., 2018, 2015; Kuo et al., 2015). For supporting vascularization of human vascular cells (a combination of endothelial cells and mesenchymal stem cells), a soft GelMA hydrogel with a compressive modulus around 2 kPa was tested to be optimal (Chen et al., 2012).

## Advantage of gelatin methacrylate hydrogel for bioengineering vascular network

The use of polymeric hydrogels is now a common practice in bioengineering vascular networks. Over the last decade, a variety of natural extracellular matrix (ECM)-derived hydrogel biomaterials have been shown to be compatible with human endothelial cell-mediated vascular morphogenesis, including Matrigel, type-I collagen, and fibrin gels (Liu et al., 2015). However, the properties of these natural ECM hydrogels are not always ideal for regenerative medicine applications. For example, Matrigel is not suitable for clinical use because it is derived from murine tumors (Aisenbrey and Murphy, 2020). Fibrin gels have limitations such as poor mechanical stability or suboptimal durability. Collagen hydrogels also have limitations in terms of extensive contraction, poor mechanical properties, and rapid degradation, all of which challenge their utilization as permanent graft material (Schneider-Barthold et al., 2016). Moreover, full polymerization of most natural ECM formulations at body temperature does not occur immediately, which may compromise gel-cell confinement if implanted in highly mobile tissues such as skeletal muscles or myocardium (Lin et al., 2013).

Conversely, some synthetic hydrogels, such as poly (ethylene glycol) diacrylate (PEGDA), have more substantial mechanical properties but inherently lack cell-responsive features, which limits their applicability in tissue engineering (Wang et al., 2021). The addition of RGD and MMP-responsive peptides had been shown to improve the biodegradability of PEGDA hydrogels and support vascularization (Moon et al., 2009). Previously, synthetic polymers and nanogels were used to encapsulate cells for *in vivo* delivery to demonstrate their safety and efficacy (Tang et al., 2017; Cui et al., 2018; Ichihara et al., 2018). Notwithstanding the benefit of these new materials, the translational usage of synthetic materials faced obstacles due to the limited data on their long-term biocompatibility. The physiological effects of their breakdown products need a thorough validation before clinical usage.

In response to these limitations, the search for improving the properties of naturally occurring ECM hydrogels has become a field of great interest. This can be achieved by chemical functionalization of ECM proteins to improve the usability of biomaterials (Tallawi et al., 2015). In this regard, GelMA is a functionalized natural (gelatin) hydrogel, and therefore shares the advantages of both natural and synthetic hydrogels. By simply modifying the degree of methacrylation, both the porosity and the degradability of GelMA hydrogel can be tuned to achieve desirable mechanical robustness without compromising cellular biocompatibility (Chen et al., 2012). These tunable mechanical properties have allowed us to fabricate GelMA hydrogels with slower *in vivo* contraction and degradation rates than collagen-type 1 gels, which are common limitations shared by the majority of natural hydrogels. Also, GelMA hydrogels are based on gelatin, which is an inexpensive denatured collagen product that can be derived from a variety of sources, making it a potentially attractive material for tissue engineering applications (Loessner et al., 2016). The presence of natural gelatin in GelMA should provide natural cell binding motifs and degradation sites, which, in principle, should facilitate cellular behavior. In addition, GelMA formulation can polymerize very rapidly (with 15 s upon exposure to UV light in the presence of a photoinitiator) and proposed that this rapid polymerization would be a critical feature to avoid hydrogel dissemination at the implantation site (Lin et al., 2013; Hong et al., 2022). Degraded products of GelMA hydrogel are simply gelatin peptides, which are parts of natural ECM components and are non-cytotoxic or non-immunogenic. More importantly, GelMA provides a permissive environment for vascular morphogenesis, making it an ideal biomaterial for bioengineering vascular networks (Lin et al., 2013).

Versatility is another advantage of GelMA hydrogels. GelMA can be used together with other natural ECM or synthetic materials to further adjust its biological or mechanical properties (Xiao et al., 2019). For example, supplementing GelMA hydrogels with fibronectin and laminin, two major basement membrane components, improves the cell spreading and migration of endothelial cells. GelMA can be mixed with other acrylated/methacrylated materials to form a photocrosslinkable co-polymers. For example, adding PEGDA or methacrylated hyaluronic acid (MeHA) into GelMA hydrogel increases the mechanical stiffness and long-term stability, making the composite hydrogels more ideal for 3D bioprinting (Kessler et al., 2017; Wang Y. et al., 2018; Velasco-Rodriguez et al., 2021). Other methacrylated materials that were tested to be comparable with GelMA include methacrylated collagen, chitosan, alginate, and dextran (Liu and Chan-Park, 2010; Kuo et al., 2015; Chuang et al., 2018; Kolawole et al., 2018; Hasany et al., 2021).

## *In vitro* application of vascularized gelatin methacrylate hydrogels

### Vascular morphogenesis in bulk gelatin methacrylate hydrogels

**FIGURE 2:**
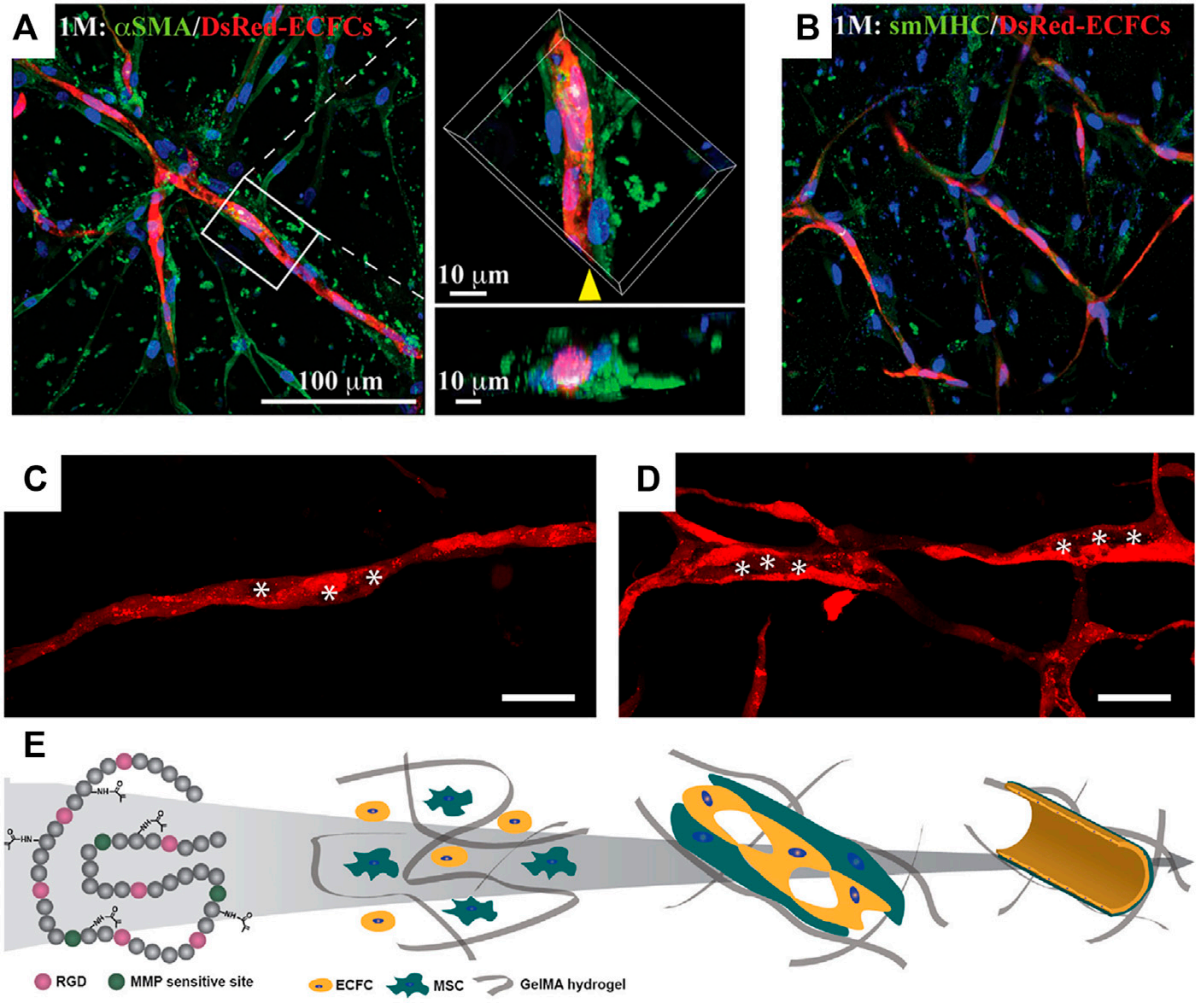
Vascular morphogenesis of human vascular progenitor cells cultured in GelMA hydrogels. Representative confocal images showing the spatial distribution of the DsRed-ECFC-lined capillaries surrounded by α-smooth muscle actin (αSMA)-expressing MSCs **(A)** and smooth muscle myosin heavy chain (sm-MHC)-expressing mature pericyte **(B)**. **(C,D)** Representative images of progressive lumen formation by DsRed-labeled ECFCs in GelMA hydrogels. The images were taken on days 3 **(C)** and 10 **(D)**. The hollow structures representing small vacuoles, fused vacuoles, and tubule compartments were labeled with asterisks. Scale bar, 100 µm. **(E)** Schematic diagram depicting the vascular network formation by human ECFCs and MSCs in GelMA. The intrinsic RGD and MMP-responsive motifs in the gelatin backbones allow cell spreading, proliferation, and migration. ECFCs and MSCs in GelMA hydrogels undergo cell organization and matrix remodeling; both are critical for vascular morphogenesis. Intracellular vacuoles form and fuse for endothelial lumen formation. MSCs differentiate into pericyte-like cells and develop perivascular coverage to stabilize the vascular networks (Chen et al., 2012).

The suitability of GelMA hydrogels for bioengineering human vascular networks was first shown in 2012 by Chen et al. (2012). Human cord blood-derived endothelial colony-forming cells (ECFCs) and bone marrow-derived mesenchymal stem cells (MSCs) were used as vascular progenitor cells. The cell-laden hydrogel was prepared by resuspending cells in a GelMA precursor solution and followed by UV photocrosslinking. In this study, GelMA hydrogels were formulated in a shape of a disk (10 mm in diameter; 200 µl in volume). Cell-laden hydrogels were cultured in a medium supplemented with pro-angiogenic factors (i.e., VEGF-A and FGF-2). In this condition, a robust formation of human vascular networks was observed after 7–10 days *in vitro* cultivation (Figure 2).

Relative soft GelMA hydrogels are ideal since the formation of vascular networks requires active cell migration and matrix remodeling. The permissive hydrogel stiffness that allows human vascular morphogenesis was determined to be 2–5 kPa in this study (Chen et al., 2012). Both endothelial and mesenchymal cell types were shown to be essential in building functional blood vessels. ECFCs form the lumens lining the capillary networks and express typical endothelial markers, CD31 and VE-cadherin (Figure 2). The presence of MSCs was crucial for the stabilization of ECFC-lined networks. α-Smooth muscle actin (αSMA)-expressing MSCs were visualized both in proximity and adjacent to the capillary structures, suggesting an ongoing process of perivascular coverage of capillaries (Figure 2A). Some MSCs differentiated into mature pericytes expressing smooth muscle myosin heavy chain (sm-MHC) (Figure 2B). Analyses of the cell-laden GelMA hydrogels at 3, 6, and 10 days of culture revealed a progressive process of lumen morphogenesis (Figures 2C,D). This process involved an initial accumulation of endothelial intracellular vacuoles on day 3 (Figure 2C), which were intracellularly arranged into rows and present in most of the ECFC-lined capillary structures. By day 6, some of the larger vacuoles had fused, forming large, intracellular luminal structures. On day 10, hollow ECFC-lined lumens were unequivocally identified and uniformly distributed in discrete locations within the capillary-like structures (Figure 2D) (Chen et al., 2012).

Established vascular networks in GelMA hydrogels still undergo active remodeling and reorganization. The microvessel densities of capillary structures can change depending on: 1) the metabolic demands of cell/gel constructs, 2) the overall oxygen tension (normoxia or hypoxia), 3) the presence of pro-angiogenic factors, 4) the maturity of endothelial cells, and 5) the durability of hydrogels (Nakatsu and Hughes, 2008; Wang et al., 2015). In general, the vascular networks in GelMA hydrogels could be maintained in regular culture conditions for 1–2 months (Chen et al., 2012). The presence of intraluminal flow (*via* medium perfusion) can further extend the durability of vascular networks (Wang X. et al., 2018).

### Microfabrication

**FIGURE 3:**
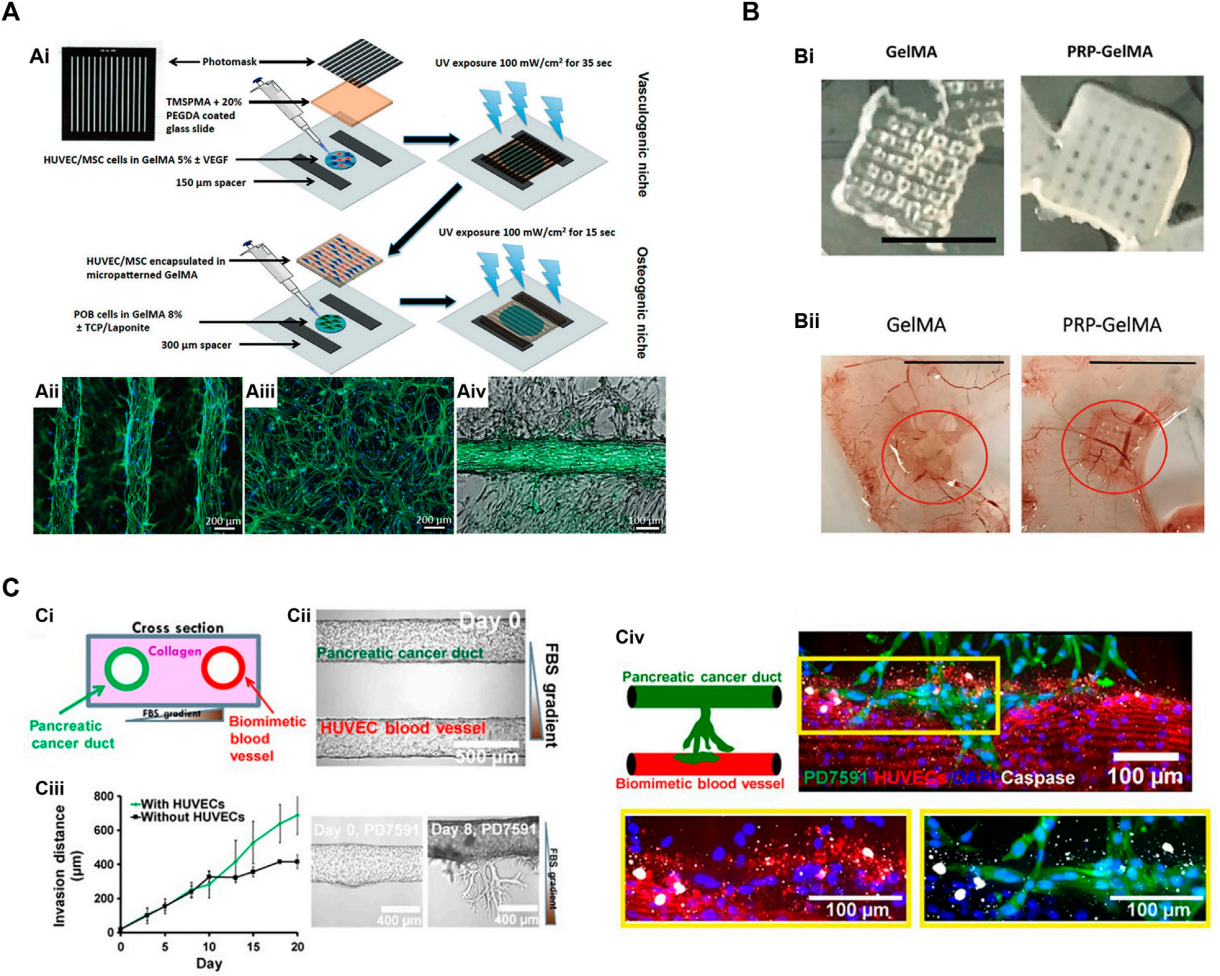
Microfabrication techniques utilize GelMA hydrogels for vascular network bioengineering. **(A)** Construction of 3D vascularized bone tissues by the photolithography technique. **(Ai)** Schematic of cell-laden micropatterned vascular networks and osteogenic niche using direct polymerization of GelMA hydrogels through photomasks (Kazemzadeh-Narbat et al., 2017). **(Aii)** Micropatterned GelMA hydrogel containing HUVECs/MSCs stained for actin filaments (green) and DAPI (blue). **(Aiii)** The same cells encapsulated in unpatterned GelMA hydrogel. **(Aiv)** Micropatterned HUVECs/MSCs forming a vascular tube surrounded by osteogenic preosteoblasts (POBs) in GelMA hydrogels. **(B)** 3D printed GelMA scaffolds incorporated with platelet-rich plasma (PRP) to enhance angiogenesis and prevent wound fibrosis (Ibanez et al., 2022). **(Bi)** Macroscopic appearance of 3D printed GelMA scaffolds with or without PRP. Scale bar = 5 mm. **(Bii)** The angiogenic properties of GelMA and PRP-GelMA scaffolds were determined by a chick chorioallantoic membrane (CAM) angiogenesis assay. PRP-GelMA scaffolds presented significantly enhanced vascularization with more recruited CAM vessels. **(C)** Microfluidic vasculature-on-a chip to study the endothelial ablation induced by aggressive pancreatic ductal adenocarcinoma (PDAC) (Nguyen et al., 2019). **(Ci)** Schematic of PDAC-on-a-chip. **(Cii)** The microfluidic device was composed of two hollow channels embedded within 3D hydrogel. One channel was seeded with endothelial cells to form a biomimetic blood vessel. The other channel was seeded with pancreatic cancer cells to form a pancreatic cancer duct. **(Ciii)** Upon cultivation, the pancreatic cancer cells began to proliferate and invade into the matrix toward the endothelial lumen. The presence of endothelial cells increased the invasion of cancer cells. **(Civ)** Cancer cell invasion resulted in endothelial apoptosis (marked by cleaved caspase-3 staining). Endothelial cells were stained with anti-CD31 antibody (in red), and pancreatic cancer cells were stained with anti-GFP antibody (in green).

Based on the success of vascularizing bulk GelMA hydrogels, microfabrication technologies were introduced to build more sophisticated engineered tissues (Figure 3). Patterned cell-laden GelMA hydrogels can be achieved by photolithography (Figure 3A). In brief, a GelMA precursor solution containing cells was photocrosslinked between two glass substrates with the spacers of desired height. UV illumination was applied through a photo mask. Only the UV exposed regions were crosslinked into hydrogels while the unexposed regions (masker areas) were removed by washing. Nikkhah et al. (2012) used this technique to create cell-laden hydrogel consisting of microconstructs and found enhance endothelial cell alignment and morphogenesis into highly organized endothelial cord structures. The same methodology had been adapted to produce defined microconstructs with variable geometries. For examples, cell encapsulated in hydrogel bricks can be fabricated with controlled sizes and cell densities (Klotz et al., 2016). These cell bricks can be used as building blocks to assemble more complicated tissues (Qi et al., 2013) or as injectable cell vehicles (Gaharwar et al., 2020). Photolithography can also apply to create build-in channels or porous networks in bulk GelMA hydrogels, aiming to improve the cell migration and oxygen/nutrient transportation in scale-up engineered tissues (Nichol et al., 2010). Interestingly, endothelial cells tend to align along the microtopologic pattern of these channels and form organized vascular networks, implying the potential of controlling the vascular patterning through fabricated microconstructs (Kazemzadeh-Narbat et al., 2017) (Figure 3A).

Cell-laden microfibers have been widely used in biomedical applications, especially in the fields of tissue engineering, 3D cell culture, and cell transplantation (Onoe and Takeuchi, 2015). In this category, GelMA microfibers can be fabricated by a microfluidic-assisted extrusion system with UV illumination [Liu et al. (2018) fabricated coaxial microfibers by co-extrusion of GelMA and alginate solutions through a concentric printhead into a CaCl_2_ solution]. GelMA served as the core (to provide a favorable 3-D microenvironment for cells) and alginate served as the sheath (to support and confine the GelMA hydrogel in the core to allow for subsequent UV cross-linking). These microfibers could be used to assemble layered microfibrous scaffold as a vascular bed by encapsulating endothelial cells in the microfibers.

GelMA has become an attractive ink material for 3D bioprinting technology due to its excellent biocompatibility, tunable rheology, and rapid crosslinking (Kim et al., 2020; Rajabi et al., 2021). The applications of GelMA in 3D bioprinting are categorized into two main types. First, GelMA can be used to prepare cell-laden microgels as modular bioinks for 3D bioprinting (Xu et al., 2020). Cell encapsulated GelMA microshperes can be produced by the microfluidic droplet generation (Liu T. et al., 2020). GelMA precursor solution flowing through a microfluidic junction with mineral oil is extruded into tiny droplets. With the aid of UV light, the microspheres were solidified (Mohamed et al., 2019). Cell embedded in GelMA microspheres are protected from the mechanical damages during 3D printing, resulting in improved cell viability.

Second, GelMA can be used directly for printing microconstructs and microchannels. Ibanez et al. (2022) printed GelMA into a porous scaffold to release pro-angiogenetic platelet-rich plasma. This 3D-printed scaffold was implanted in mice to demonstrate an enhanced angiogenic potential while not inducing fibrosis (Figure 3B). Nulty et al. tested several bioinks (i.e., alginate, fibrin, and GelMA) to produce prevascularized bone grafts containing human endothelial cells and bone marrow stromal cells (Nulty et al., 2021). Only fibrin and GelMA hydrogels allowed the formation of vascularized constructs. Implantation of vascularized bone grafts in rat femoral defects induced bone regeneration and achieved bone healing in 12 weeks. Jia et al. designed a multilayered coaxial nozzle to print a formulated cell-responsive bioink consisting of GelMA, sodium alginate, and 4-arm poly (ethylene glycol)-tetra-acrylate (PEGTA) to create perfusable hollow tubes. The printed structures were built sequentially by ionic crosslinking of alginate and covalent crosslinking of GelMA/PEGTA with Irgacure 2959 and UV irradiation. The alginate parts were removed by EDTA treatment, leaving a highly porous hydrogel scaffold. This scaffold provided a favorable microenvironment that supported the proliferation and early maturation of gel-encapsulated vascular cells as well as the bioprinting of complex 3D vasculature.

GelMA with different mechanical strengths (achieved by the degree of methacrylation or photocrosslinking time) can be integrated in one 3D printed construct to biomimetic the properties of biological tissues. For example, Byambaa et al. (2017) utilized an extrusion-based direct-writing bioprinting strategy for fabricating perusable vascularized bone construct. Cell-laden GelMA cylinder rods were deposited layer-by-layer to mimic bone-like architecture. The inner core containing a mixture of human endothelial cells and MSCs was printed with a GelMA hydrogel with low stiffness. The fast degradation of the inner core hydrogel allowed for forming perfused central lumen. The inner core was surrounded by GelMA cylinder rods of higher mechanical stiffness, which match the mechanical environment in bone matrix. A gradient of VEGF proteins and silicate nanoparticles was established during bioprinting to guide the angiogenesis and osteogenesis. Increasing angiogenic or osteogenic gene expressions such as CD31, Col1, ALP, OCN, and OPN was observed in this bioprinted bone scaffold.

To improve the structural fidelity of 3D bioprinting, Ouyang et al. (2020) recently developed the void-free 3D printing (VF-3DP) method. In this process, a templating bioink (i.e., 7.5 wt% gelatin) was printed side by side with a biocompatible matrix bioink (i.e., 5% GelMA), followed by photocrosslinking of the whole constructs. The gelatin template was washed out by incubating the printed materials at 37°C, leaving the GelMA hydrogel reminded. Importantly, human endothelial cells can be encapsulated in the GelMA hydrogel with high cell viability. This study demonstrated the bioprinting of hydrogel-based microfluidics with customized flow patterns and endothelialization, with the potential to fabricate self-supported perfusable hydrogel constructs.

### Microfluidic vasculature-on-a chip

ECM hydrogels have been used as matrix to bioengineer microvascular networks in the cell culture chambers or channels in microfluidic devices (Smith and Gerecht, 2014). Successfully vascularized chip devices can be used for drug screenings and are sometimes referred to as “vasculature-on-a chip” (Franco and Gerhardt, 2012; Kim et al., 2017; Tomasina et al., 2019; Henderson et al., 2020). In this application, the usage of GelMA allowed the investigators to tune the matrix stiffness, which critically regulates the diameters, densities, and barrier functionalities of capillary networks (Chen et al., 2012).

Vascularization in microfluidic-based platforms is commonly achieved by polydimethylsiloxane (PDMS) device (Kim et al., 2017; Phan et al., 2017). In general, a vascular chamber is fabricated by PDMS material with inlet and outlet channels. Vascular-forming cells are loaded with hydrogels into the vascular chamber. The device is connected to an external pump to circulate oxygenized culture medium. Within 7–10 days, vascular cells assemble into vascular networks with interconnective lumens linked to the inlet and outlet channels, resulting in the medium perfusion inside the vascular lumens (Mandrycky et al., 2021). Across all on-chip models, the key features are providing continuous physiological flow and precisely controlling the input and output components. The microfluidics can be designed with variable geometries or multiple chambers to mimic physiological or pathological conditions. For example, Nguyen et al. established a coculture of cancer cells in a chip-based vascular network to study the mechanism of tumor vascular invasion (Figure 3C) (Nguyen et al., 2019). However, since the PDMS materials are not biodegradable, these PDMS-made vascular devices cannot be used to generate engineered tissue constructs for transplantation.

Perfusable vasculature with complex hierarchical networks can be fabricated by using sacrificial templates. Miller et al. (2012) demonstrated the fabrication of 3D structures made from 3D printing of sugar glass scaffolds. The scaffolds were embedded in a cell-laden hydrogels (i.e., GelMA). The sacrificial template print was then dissolved and flushed with water, leaving a patterned channel network with perfusable lumens (Miller et al., 2012). Cells can then be seeded on the wall of the channels, creating an endothelialized vascular bed. This method is notably attractive due to its flexibility in accommodating a variety of stromal/parenchymal cell type as well as ECM material. Other sacrificial materials have improved on this initial work, but the overall workflow is similar (Bertassoni et al., 2014; Kolesky et al., 2014). Recently, Kinstlinger et al. (2021), Kinstlinger et al. (2020) improved the fabrication by using laser-sintered carbohydrate powders which allows the printing of more complicated 3D sacrificial structures. Kolesky et al. (2014) utilized sacrificial printing to generate an interconnected vascular network that perfuses throughout a thick (centi-meter scale) engineered construct of human cells. The build-in channel network improves cell proliferation and cell metabolism through fluid convection (Kolesky et al., 2016). These scalable strategies are ideal for the fabrication, perfusion culture and volumetric analysis of large tissue-like constructs with complex and hierarchical vascular architectures (Song H. et al., 2018; Redd et al., 2019).

## *In vivo* application of gelatin methacrylate hydrogels for therapeutic vascularization

Recent tissue engineering methods have been applied to a variety of diseases, including cardiovascular disease, bone disease, and neuronal disease (Carmeliet, 2003; Rouwkema et al., 2008; Lovett et al., 2009). Especially, GelMA hydrogel has been developed as a promising strategy for therapeutic cell delivery to injury sites due to its high biocompatibility. Neovascularization is critical for the onset of the regenerative process (Rodrigues et al., 2019). The new circulation in healing wounds establishes a pro-regenerative niche and brings in the supplement of oxygen/nutrients, as well as stem/progenitor cells and supporting cells from both bone marrow and surrounding tissues. In this regard, GelMA was reported in several publications to be an ideal vehicle to deliver vascular progenitor cells and pro-angiogenic factors for *in vivo* vascularization. Over the past decades, various cell types, including human umbilical vein endothelial cells (HUVECs), MSCs, adipose-derived stem cells (ADSCs), and ECFCs within GelMA hydrogel have proved to increased therapeutic effects on cardiovascular diseases compared to those without the hydrogel system.

### Transplantation of vascularized cell-laden gelatin methacrylate hydrogels

The success of *in vitro* vascular formation in hydrogel constructs does not guarantee the grafts will anastomose with the blood vessels and achieve perfusion by host circulation after *in vivo* transplantation. The properties of implant materials must be considered, including their immunogenicity, biodegradability, and the tendency to provoke foreign body reactions (Anderson et al., 2008; Song R. et al., 2018). Chen et al. (2012) first demonstrated the suitability of a photopolymerizable GelMA hydrogel to support *in vivo* vascularization of human vascular networks. Surgically implantation of prevascularized GelMA hydrogels (i.e., vascular networks assembled by human ECFCs and MSCs) into immunodeficient mice results in a rapid formation of functional anastomoses between the bioengineered human vascular network and the mouse vasculature. Histological examination carried out by hematoxylin/eosin (H&E) staining revealed numerous human blood vessels containing mouse erythrocytes throughout the GelMA grafts. Furthermore, it is shown that the degree of methacrylation of the GelMA can be used to modulate the cellular behavior and the extent of vascular network formation. Soft GelMA grafts allowed a uniformly distributed vascularization, while microvessels were mainly restricted to the periphery of the constructs made of stiffer GelMA hydrogels. Meanwhile, non-perfused human-ECFC-lined capillary-like structures were mainly observed in the center of implants, suggesting an ongoing process of connection between the human preformed capillaries and the mouse perfused vessels coming from the surrounding host tissues. GelMA hydrogels that are too stiff will delay the in-growth of host vessels, resulting in poor vascularization and necrosis in bulk implants. Build-in microchannels or porous structures might be considered for the scaling up of bioengineered tissue grafts.

The recruitment of host myeloid cells is necessary in ECFC/MSC-mediated neovascularization (Melero-Martin et al., 2010). Two types of myeloid cells, neutrophils and macrophages, were determined to be critical mediators of graft vascularization. Non-inflammatory host neutrophils were recently found to be indispensable for vascularization through the secretion of pro-angiogenetic factors, modulation of the inflammatory response, and remodeling of the ECM matrix (Lin et al., 2017). Tissue macrophages were known to promote angiogenesis and mediate vascular anastomosis between host and graft vessels (Fantin et al., 2010). Lin et al. (2013) showed that the interaction of host myeloid cells with the cell-laden GelMA constructs was significantly altered by the degree of GelMA polymerization. Significantly less penetration (but more accumulation in the boundary) of both murine neutrophils and macrophages in constructs that were crosslinked for a more extended period. This is important because accessibility of host cells is critical for ECFC-mediated vasculogenesis, sprouting of host microvessels *via* angiogenesis, and ultimately formation of anastomosis between bioengineered vessels and host sprouts.

### *In situ* photocrosslinking of gelatin methacrylate hydrogels

**FIGURE 4:**
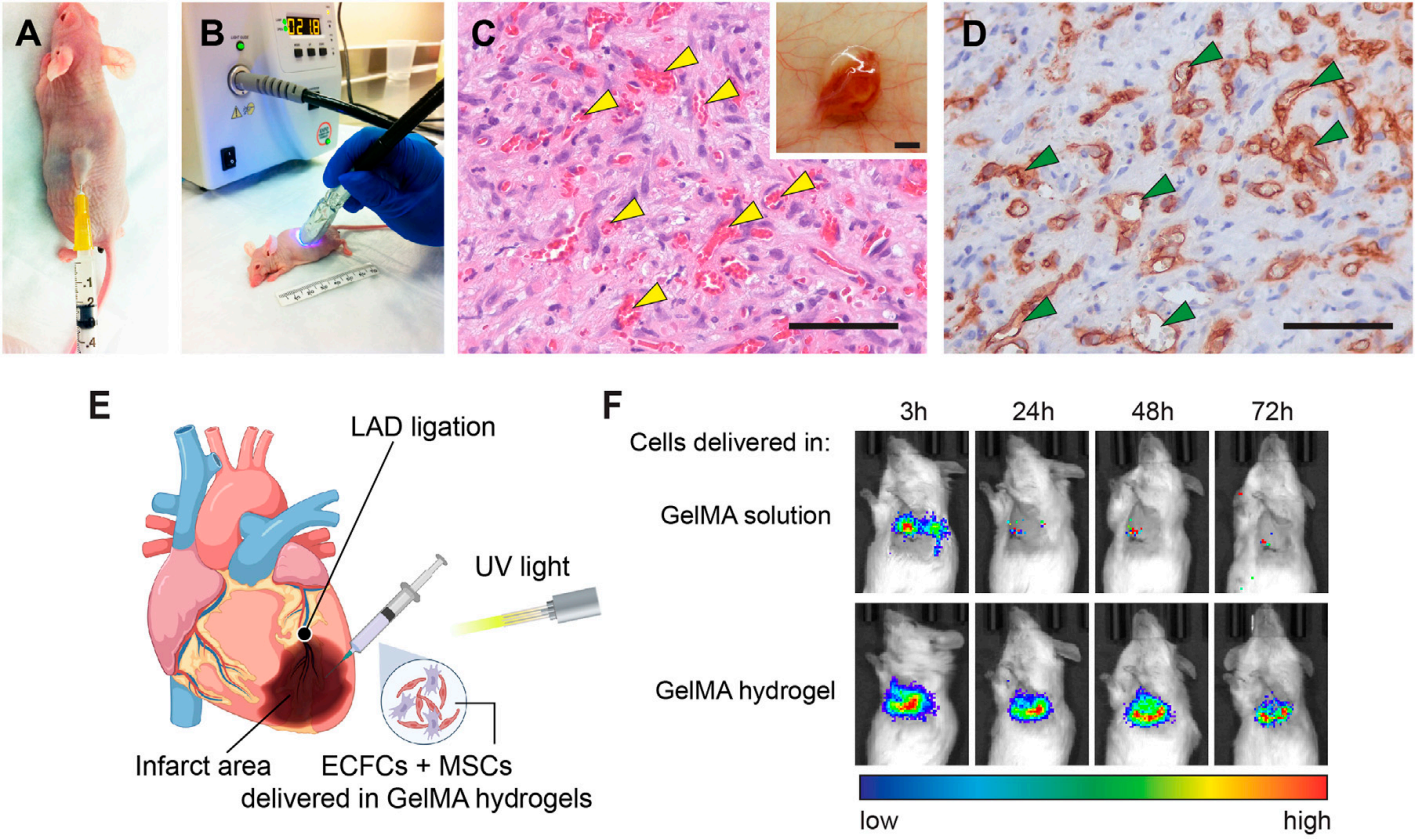
Transdermal and intramyocardial photopolymerization of GelMA hydrogels for *in vivo* vascular network bioengineering. **(A)** Human ECFCs and MSCs were resuspended in a GelMA precursor solution and injected subcutaneously into nude mice. **(B)** Representative images of a mouse receiving transdermal UV light. **(C)** Representative H&E-stained section from a day 7 construct that was transdermally polymerized. Yellow arrowheads mark perfused blood vessels. Scale bar, 50 µm. Insert: the vascularized construct in the subcutaneous space after 7 days. Scale bar, 500 µm. **(D)** Human ECFC-formed vascular networks were identified by the human-specific CD31 immunohistochemistry. Green arrowheads mark perfused lumens lined by hCD31^+^ ECFCs. Scale bar, 50 µm (Lin et al., 2013). **(E)** Schematic depicting the intramyocardial cell injection. Myocardial infarction was surgically induced by left anterior descending artery (LAD) ligation. Human vascular progenitor cells (ECFCs + MSCs) resuspended in GelMA precursor solution were injected and photocrosslinked into a cell-laden hydrogel. **(F)** Viable cell retention was measured by bioluminescence imaging of human ECFCs expressing a luciferase reporter. The results showed that the GelMA hydrogel significantly enhanced cell retention in ischemic hearts during the first 72 h (Hong et al., 2022).

*In situ* polymerization of cell-laden GelMA hydrogels following its injection *in vivo* has been demonstrated by Lin et al. (2013). They performed the transdermal polymerization of GelMA through the skins of mice and pigs (Figure 4). The light can be transmitted (15–40% transmission) through the skin of nude mice using a UV lamp system with a wavelength between 320 and 500 nm. This spectrum includes blue and violet visible light (380–500 nm) and UVA light (315–400 nm), but not more harmful UVB and UVC light. They found that the skin of mice after 15–90 s UV exposure had a similar number of apoptotic cells as compared to the skin of unexposed mice. Meanwhile, the GelMA hydrogels can be polymerized by transdermal UV illumination for only 15 s. Therefore, they concluded that the operating range of UV light exposure required for *in situ* photopolymerization was deemed sufficiently safe.

Human ECFCs and MSCs delivered by transdermal polymerization of GelMA achieved a robust formation of vascular networks *in vivo* (Figures 4C,D) (Lin et al., 2013). They modulated the vascularization process *via* adjusting the initial UV exposure time (15–45 s range) and found that increased GelMA crosslinking decreased the vascular density and average lumen size. Compared with the surgical implantation of *ex vivo* cell-laden GelMA, enabling hydrogel injection and *in situ* polymerization would be more desirable because 1) it would eliminate the need for surgical incisions, 2) enable the cell-laden hydrogel solution to acquire complex shapes and precise dimensions *in situ*, prior to polymerization, and 3) improve intimate contact of the polymer with the surface micro-roughness of the tissue at the implantation site. Furthermore, a recent study demonstrates that implants containing assembled human vascular networks fail to engraft efficiently owing to their inability to engage non-inflammatory host neutrophils upon implantation into mice (Lin et al., 2017). By contrast, injecting the unassembled single cell suspension of vascular progenitor cells the achieved better vascularization by actively engaging host neutrophils. The same result was also observed by Lin et al. (2013) showing that the transdermal polymerization of cell-laden GelMA formed high vascular densities than the transplantation of premade GelMA constructs.

Rapid *in situ* photocrosslinking of GelMA hydrogels is ideal for delivering therapeutic cells into constantly moving tissues and organs, like the skeleton muscles and hearts. Our recent study demonstrated the application of GelMA to deliver human vascular progenitor cells into ischemic heart muscles (Hong et al., 2022). Low cell retention and engraftment are the major obstacles to achieving a significant functional benefit following cell-based therapy for myocardial infarction (MI) (Li et al., 2021). Given that the heart is a contractile pump, beating hearts actively push therapeutic cells injected in liquid vehicles out of the myocardium, resulting in a rapid loss of cell retention (Teng et al., 2006; Terrovitis et al., 2009; Vrtovec et al., 2013; Menasché, 2018; Crisostomo et al., 2019; Liu Z. et al., 2020; Li et al., 2021). In our study, the intramyocardial injection of human ECFCs and MSCs resuspended in a GelMA precursor solution followed by transmyocardial UV illumination, resulting in an *in-situ* photocrosslinked hydrogel that effectively retains the cells inside the ischemic myocardial tissue (Figure 4E) (Hong et al., 2022). This approach maintained high viability and cell retention, providing a significant advantage over cells injected in liquid or unmodified ECM gels (Figure 4F). We demonstrated that the beneficial myocardial remodeling and stabilization of cardiac functions post-MI was enabled by the engrafted cells *via* the engagement and polarization of host pro-regenerative neutrophils through TGFβ signaling. Regarding the translational potential, the cell/gel mixture can be delivered through a minimally invasive catheter-mediated procedure. In human patients, this method can be achieved by a long catheter threaded through the peripheral blood vessels into the left ventricle, where the cells are injected through the endocardium (Suncion et al., 2014). In general, this approach is less invasive because it could avoid open-chest surgery. Also, UV light illumination could be delivered through a fiber-optic system together with the catheter (Roche et al., 2015).

### *In vivo* delivery of pro-angiogenic agents

GelMA hydrogels have been used to encapsulate organic or inorganic compounds to improve therapeutic effectiveness in vascular diseases (Briquez et al., 2016; Ibanez et al., 2022). These factors could be therapeutic proteins, bioactive lipids, metabolites, and extracellular matrix elements (Kurian et al., 2021; Chen et al., 2022; Tang et al., 2022). For example, Quint et al. (2021) performed *in vivo* printing of GelMA-based scaffolds composed of VEGF-bound Laponite. Laponite nanoclay was used to deliver pro-angiogenic proteins for sustained slow release. The GelMA bioink was extruded through a handheld printer with a UV LED light to fill volumetric muscle loss injuries. Neovascularization followed with new myofiber formation was observed. Zhang et al. (2021) synthesized a photocrosslinked decellularized amniotic membrane (dHAMMA) and blended it with GelMA hydrogels. This GelMA-dHAMMA composite hydrogel was used as biomimetic skin-substitute tissues for slow releasing pro-regenerative factors from decellularized matrix. They successfully demonstrated that GelMA-dHAMMA promoted angiogenesis and fibroblast proliferation, resulting in accelerated wound healing. Yan et al. (2021) incorporated a microbial lipopeptide, called surfactin, in GelMA hydrogel to treat diabetic wounds. Surfactin increased the mechanical properties of GelMA hydrogel and accelerated diabetic wound healing *via* regulating macrophage polarization and promoting angiogenesis. Recently, Tang et al., 2022 created a GelMA patch to deliver MSC-derived extracellular vesicles (EVs) to ischemic heart muscles. EVs-laden GelMA had a remarkably therapeutic effect in a myocardial infarction mouse model. This hydrogel inhibited apoptosis of cells in the infarct zone and increased angiogenesis and myogenesis. Overall, the applications based on GelMA hydrogels to release therapeutic agents are numerous. Many of these studies target the acute wound healing process, including angiogenesis, inflammation, and fibrogenesis, and aim to modulate a pro-regenerative microenvironment through bioengineering approaches.

## Conclusion and perspectives

Since the first synthesis of GelMA by Nichol et al. (2010), the usage of GelMA hydrogels in biomedical applications has advanced rapidly. From 2013 to 2021, more than one thousand GelMA-related papers were published (based on Pubmed search), and the number of publications increased annually. The popularity of GelMA is mainly due to its excellent biocompatibility and biodegradability, making it ideal for various biomedical applications. Meanwhile, GelMA integrates well with the growth of new technologies like 3D printing and microfluidics, resulting in many exciting progresses. Over the last decade, GelMA has been adapted to applications in almost all aspects of tissue engineering and regenerative medicine by targeting various types of tissues and organs. Here, we summarized trends followed by investigators with regard to the use of GelMA hydrogels for bioengineering human vascular networks. We highlighted the design principles to make GelMA hydrogels suitable for vascular morphogenesis based on our knowledge of vascular biology. We also emphasize the key factors that govern the functional vascularization of GelMA-based vascular constructs after transplantation *in vivo*. Potential topics that we briefly mentioned but are worth further investigation are the development of visible light-based photocrosslinking systems (Noshadi et al., 2017; Sharifi et al., 2021) and the advances in the GelMA-based composite materials (Kurian et al., 2021; Alexa et al., 2022; Liang et al., 2022). In addition, research efforts will continue on the multiplex functionalities of GelMA-based biomaterials. More advanced GelMA-based hydrogels aiming for clinical translations can be expected to be available in the near future.
